# SHiNeMaS: a web tool dedicated to seed lots history, phenotyping and cultural practices

**DOI:** 10.1186/s13007-020-00640-2

**Published:** 2020-07-23

**Authors:** Yannick De Oliveira, Laura Burlot, Julie C. Dawson, Isabelle Goldringer, Darkawi Madi, Pierre Rivière, Delphine Steinbach, Gaëlle van Frank, Mathieu Thomas

**Affiliations:** 1 Université Paris-Saclay, INRAE, CNRS, AgroParisTech, GQE-Le Moulon, 91190 Gif-sur-Yvette, France; 2 AgroBioPerigord, 24000 Périgueux, France; 3 Department of Horticulture, University of Wisconsin–Madison, Madison, WI 53706 USA; 4 Réseau Semences Paysannes, 47190 Aiguillon, France; 5 CIRAD, UMR AGAP, 34398 Montpellier, France; 6 Univ Montpellier, CIRAD, INRAE, Montpellier SupAgro, Montpellier, France

**Keywords:** Database, Plant breeding, Software

## Abstract

**Motivation:**

In 2005, researchers from the French National Research Institute for Agriculture, Food and Environment (Institut national de recherche pour l’agriculture, l’alimentation et l’environnement, INRAE) started a collaboration with the French farmers’ seed network Réseau Semences Paysannes (RSP) on bread wheat participatory breeding (PPB). The aims were: (1) to study on-farm management of crop diversity, (2) to develop population-varieties adapted to organic and low-inputs agriculture, (3) to co-develop tools and methods adapted to on-farm experiments. In this project, researchers and farmers’ organizations needed to map the history and life cycle of the population-varieties using network formalism to represent relationships between seed lots. All this information had to be centralized and stored in a database.

**Results:**

We describe here SHiNeMaS (Seeds History and Network Management System) a web tool database. SHiNeMaS aims to provide useful interfaces to track seed lot history and related data (phenotyping, environment, cultural practices). Although SHiNeMaS has been developed in the context of a bread wheat participatory breeding program, the database has been designed to manage any kind and even multiple cultivated plant species. SHiNeMaS is available under Affero GPL licence and uses free technologies such as the Python language, Django framework or PostgreSQL database management system (DBMS).

**Conclusion:**

We developed SHiNeMaS, a web tool database, dedicated to the management of the history of seed lots and related data like phenotyping, environmental information and cultural practices. SHiNeMaS has been used in production in our laboratory for 5 years and farmers’ organizations facilitators manage their own information in the system.

## Background

Due to a lack of effort in breeding varieties adapted to organic and low-input agriculture [1] farmers currently have limited access to the proper crop diversity. Cultivated biodiversity has declined in the landscape for decades as a result of the industrialization of agriculture [2]. For these reasons, there is a need to deploy a cultivated diversity adapted to diverse practices and contexts.

In order to create diverse heterogeneous varieties of bread wheat adapted to farmers’ practices and needs, researchers from the French National Research Institute for Agriculture, Food and Environment (INRAE) and the wheat group of the French farmers’ seed network Réseau Semences Paysannes (RSP) started a collaborative program in 2005. The aims of this program are (i) to study on-farm management of crop diversity [3], (ii) to develop population-varieties adapted to organic and low-inputs agriculture in the context of a participatory plant breeding program involving a collective of farmers, facilitators and researchers [4, 5] and, (iii) to co-develop methods and tools such as data management tools, experimental designs and statistical methods adapted to on-farm experiments [6, 7], in order to foster genetic and social innovations.

**Fig. 1:**
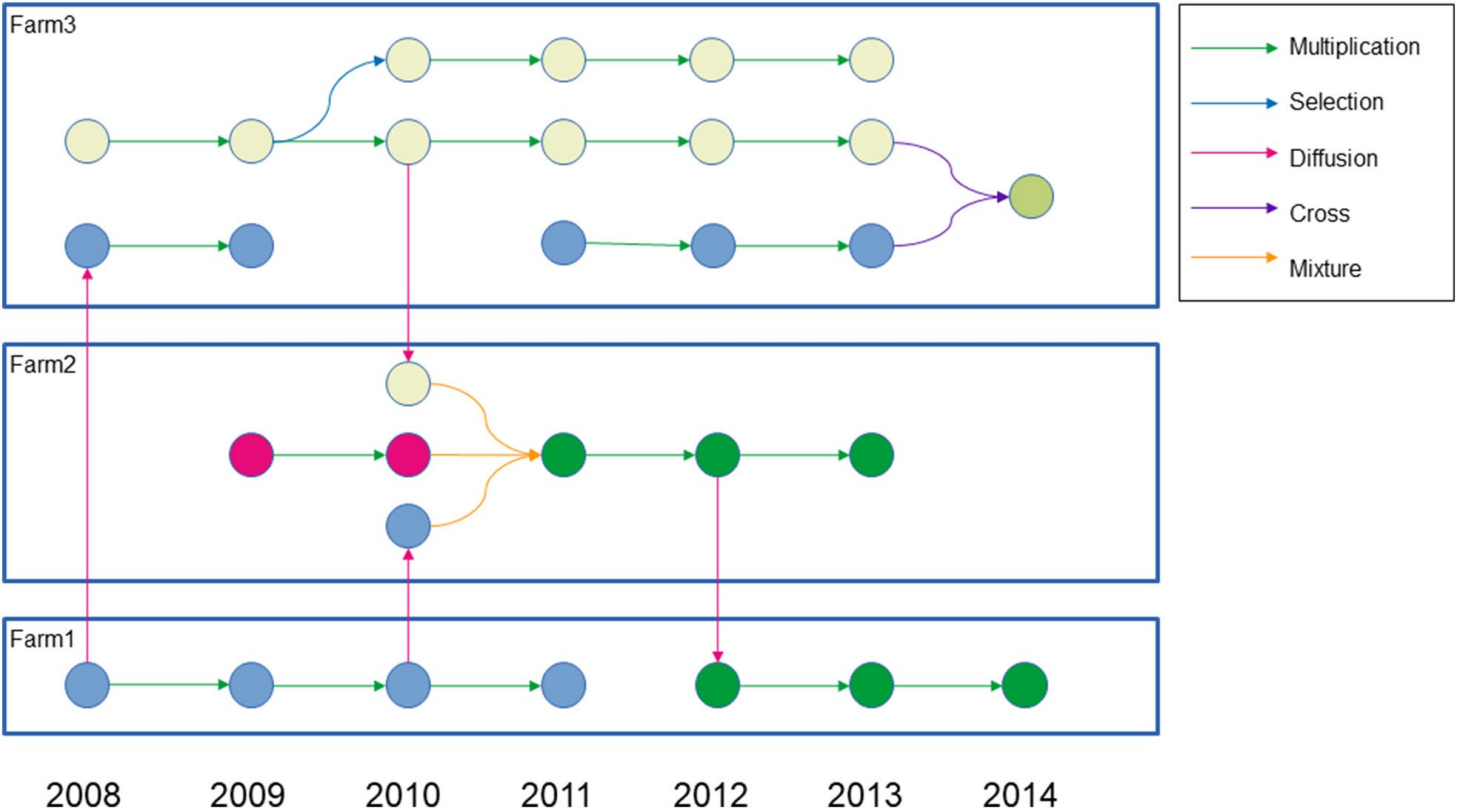
Seed lots network.. A seed lot is represented by a colored plain circle. A different color means a different germplasm. A relation is depicted by an arrow whose color indicates the type of relation

In this context, participants have (i) developed, evaluated, and measured at global and individual levels new population-varieties [6, 8, 9] and (ii) fostered seed and knowledge circulation among participants in a large network of farms (more than 100 farms in 2018). This program therefore produces a lot of data, and, in particular, relational information between seed lots (traceability) that can be represented as a network (Fig. 1). Thus several dozen users involved in the program, the researchers and facilitators needed a tool adapted to the monitoring of decentralised experimentation, accessible online and with user-friendly interfaces, to facilitate the management of the data produced. Moreover, it is important for us to develop a tool distributed under a free licence, free of charge and using free technologies.

Some tools we identified like PlantDB [10], Phytotracker [11] or SeedUSoon [12] manages seed lots and their stock information but doesn’t fit our requirements as they are not adapted to field experimentation. For instance, PlantDB is mainly related to genotyping data management but doesn’t provide a system to collect data from the field. Phytotracker provides features to design plant crosses but no additional data (trait description) can be recorded. Moreover, Phytotracker and PlantDB use proprietary technologies and are operating system dependant. SeedUSoon fill this technological gap as it uses Java and MySQL DBMS, but it offers a limited number of scenarios. For instance, mixture, diffusion or selection can’t be handled in this tool. Finally, none of them provide feature to load data from a file, a mobile app or a web application making these tools unsuitable for field experimentation. Another one, GnpIS [13], manages seed lots and germplasm, but doesn’t ensure seed lots traceability, it doesn’t provide interfaces for data management and consequently, can’t be considered as a data management tool. The last, the Breeding Management System [14] provides a lot of useful features but does not ensure complete traceability of seed lots and a part of its component are under proprietary licence. To fill the gaps in these tools and to avoid expensive work to improve one of them to fill our requirements, we developed SHiNeMaS (Seeds History and Network Management System), a web tool to manage the network and history of seed lots and related data.

## Implementation

SHiNeMaS has been developed using the Python language [15] and the Django framework^1^, a free and open source Python web framework. Django provides an API to interact with the database and migration tools that generates scripts automatically when the relational model is updated. It is an important feature as we aim to develop and distribute further versions of the tool. SHiNeMaS is deployed on GQE-Le Moulon’s server with PostgreSQL DBMS, but, according to Django’s API, MySQL, Oracle and SQLite DBMS can also be used. SHiNeMaS is distributed by two way, (i) a Docker container which makes possible to use the software as a desktop application or a server based application, but it works only with Linux, (ii) the code source which will need IT knowledge to deploy in your web server (often Apache or Nginx). As for any tool using a database, it is important to respect backup rules to avoid any data loss. In any case a documentation is provided to install and deploy SHiNeMaS.

## Results and discussion

SHiNeMaS has been created to support any kind of cultivated species such as cereals, fodder and vegetable plants. The web application has been designed to satisfy the needs of researchers, farmers’ organization facilitators, farmers and gardeners. Data access is protected by a system of user accounts with two levels of authorization: read/write user for data management and read-only user for data querying.

### Database structure

The SHiNeMaS relational schema is provided as Additional file 1. The first part of the schema describes the genetic material. *Seed_lot* is the main table of the schema while *Update_Quantity* stores stock inventory events. Quantities are managed using gram as the unit. A seed lot is linked to the tables *Germplasm* and *Location*. The table *Germplasm_type* manages the type of the genetic material. SHiNeMaS offers a flexible way to manage inbreed lines, hybrids, populations or any kind of genetic material you want to describe. The name of the species is also an attribute of the genetic material.

A second part of the schema describes the network of participants in the breeding program. The table *Person* describes the actors of the network, it can be farmers, facilitators, researchers etc. while the table *Location* describes an experimentation site, an institute, or any legal entity.

A third part of the schema represents relations between seed lots with the *Relation* table. Each relation belongs to an event depicted in the tables *Reproduction, Selection, Diffusion* and *Mixture*. A relation is basically a double link to the *Seed_lot* table with one seed lot considered as a parent and the other one as a child. The quantity (in grams) used for the parent seed lot in a relation is stored in this table. This way to manage relations between seed lots ensures the ability of the tool to design any kind of crosses as long as they are described step by step. For instance, a back cross can be defined this way : AxB $\rightarrow$ C in a first step, then CxB to ensure the backcross or a poly-cross can be handled like that: AxB $\rightarrow$ C, DxE $\rightarrow$ F then FxC to ensure the poly-cross. Open pollinated event AxBxCxD $\rightarrow$ E can be handled with a mixture.

The last part of the schema represents the data (*Raw_data* table) related to the *Relation* table or to the *Seed_lot* table. Each data is described according to a variable and a method.

### Web application features

#### Manage data with files

**Fig. 2:**
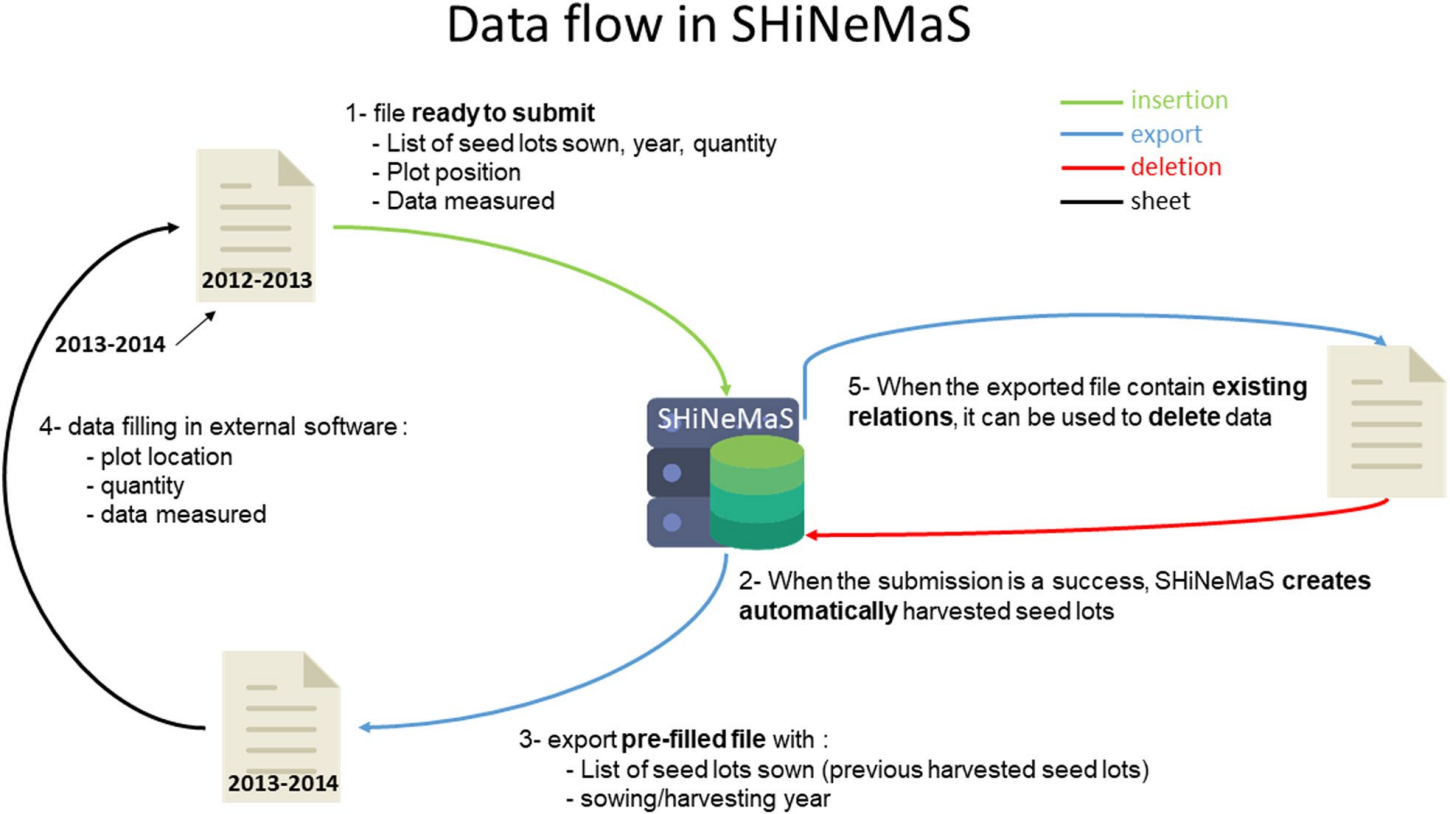
Data flow in SHiNeMaS. SHiNeMaS has been designed to work efficiently with files. When submitting a file (1) the application will create the resulting seed lots for each event (2). These seed lots can be used later to create and export a new file (3). Thus, the user only need to fill the remaining cells in a spreadsheet software (4) before a new submission. A user can also export a file with recorded relations and use it to run a deletion process (5)

SHiNeMaS provides interfaces to load (Fig. 2, step 1) relational information between seed lots and the associated data concerning phenotypes, environments and cultural practices with tabulated format files. SHiNeMaS accounts for five kinds of events to describe the type of relationship between seed lots: diffusion, reproduction, cross, mixture and selection resulting in five files format with specific and mandatory headers. A sixth file format describes individual measures. In addition to the mandatory headers, users can add headers corresponding to the variables measured (phenotyping, environment description, cultural practices). The associated data submitted are linked to a relation, a sown seed lot, a harvested seed lot etc. according to the type of the variable and the type of event. A date can be associated to each measure respecting the ISO 8601^2^ standard (YYYY-MM-DD). The format DD/MM/YYYY is also accepted in SHiNeMaS.

After each file submission, a report is displayed to inform the user whether the data has been successfully inserted into the database and, if not, what was wrong with the file. If the submission is valid, SHiNeMaS will create automatically the output seed lot of each relation (Fig. 2, step 2, for example, the harvested seed lots of a reproduction) with a specific pattern to create the name of the resulting seed lot.

Currently, SHiNeMaS does not provide any native connection to mobile devices. However, the tool we describe here uses tabulated text files to record data. This kind of file can be easily transferred to any mobile device and can be filled with usual editors on this device; In this case offline mode is not a problem as all information needed is stored in the file and accessible in offline mode.

Also, a reproduction file can be used to delete (Fig. 2, step 5) a set of reproduction events. These events can be deleted alone or in cascade, which means that the event itself, all the subsequent events and all the data are deleted.

#### Prepare files with wizard tools

Because preparing a large file in a spreadsheet software can be a fastidious task, SHiNeMaS provides a helpful assistant to the creation of files using the seed lots or relations already recorded in the database (Fig. 2, step 3). By executing successive queries from a simple form, a user can build a list of seed lots or relations and export it in one of the file format described below. Thus, the user will only have to fill the remaining cells (Fig. 2, step 4) before the next submit.

#### Data management with single form

**Fig. 3:**
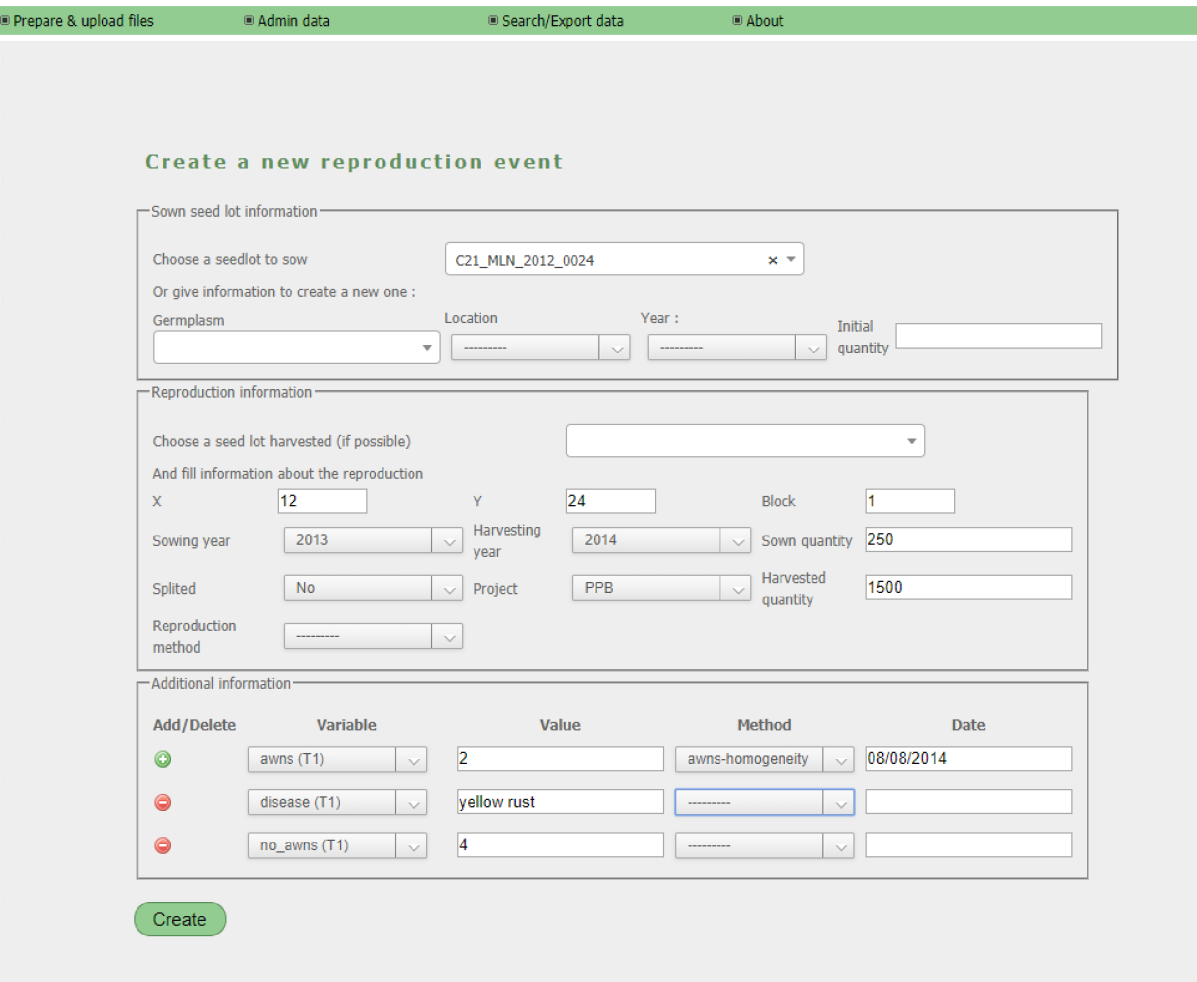
Reproduction creation form. Form to create a new reproduction. The form is divided in three fieldsets. The first one requires sown seed lot information. The second requires to fill the reproduction information. The last one contains the data related to the reproduction

SHiNeMaS provides form interfaces to create single relation events (Fig. 3 shows a form to create a new reproduction event). As for the files, a different form has been designed for each event type. When creating a new single event, the user can also add all the data measured, like when submitting a file. Forms are also provided to manage any other type of information recorded in the database (projects, variables, methods, etc.).

#### Data querying

SHiNeMaS provides query interfaces to retrieve information about seed lots such as (1) parentage, (2) generation number (on the current location and in total) and the quality of information, (3) data regarding variables measured during a given period of time. The query can be filtered according to various criteria and the results are displayed in three tables showing the data related to seed lots, the data related to the relations and individuals data.

**Fig. 4:**
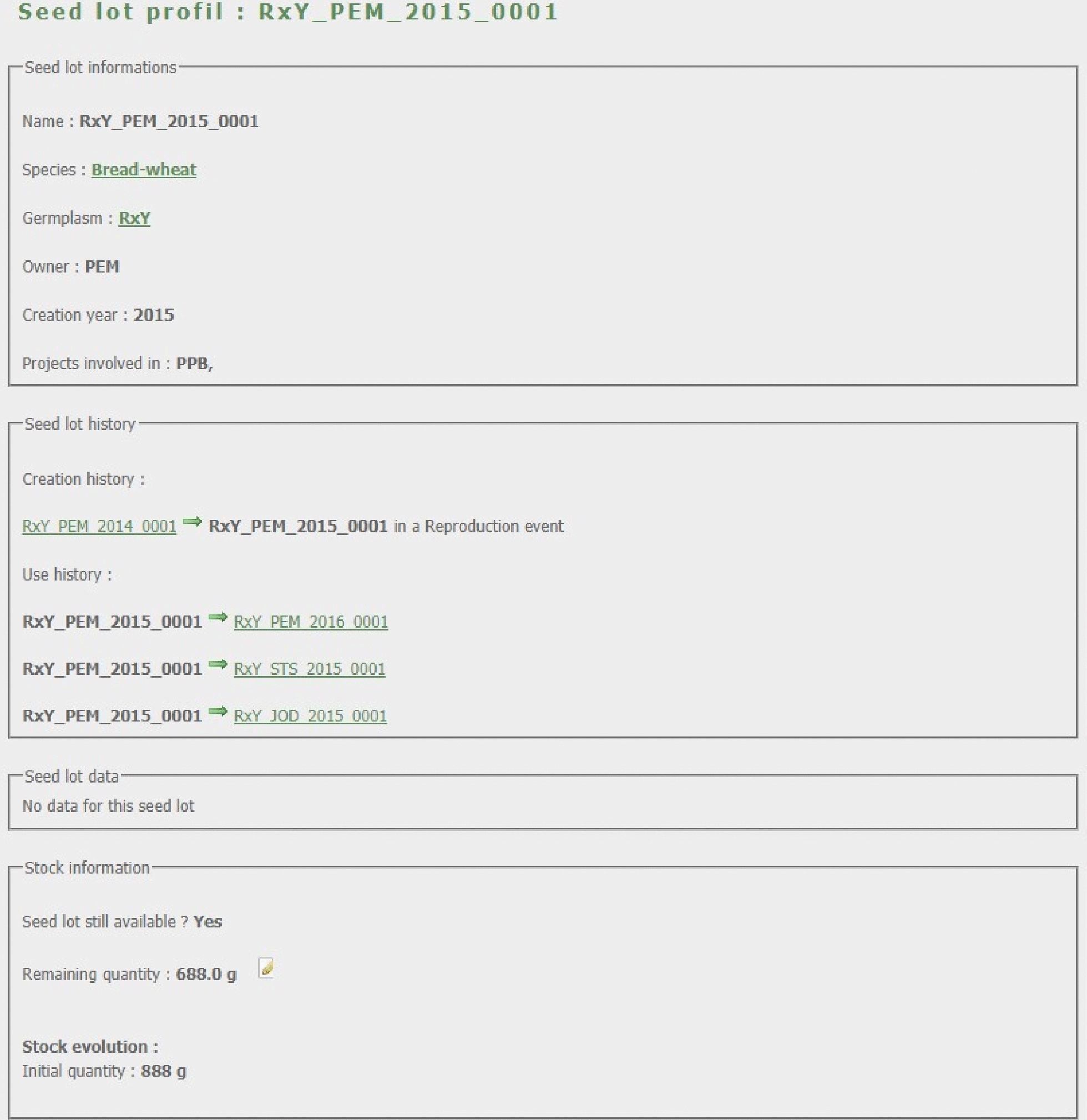
Seed lot card. The seed lot card is divided in four frames: (1) The seed lot information with its name, species, germplasm etc. (2) The history of the seed lot, how it has been created and how it has been used. (3) The data linked to the seed lot. (4) The stock level and change history

**Fig. 5:**
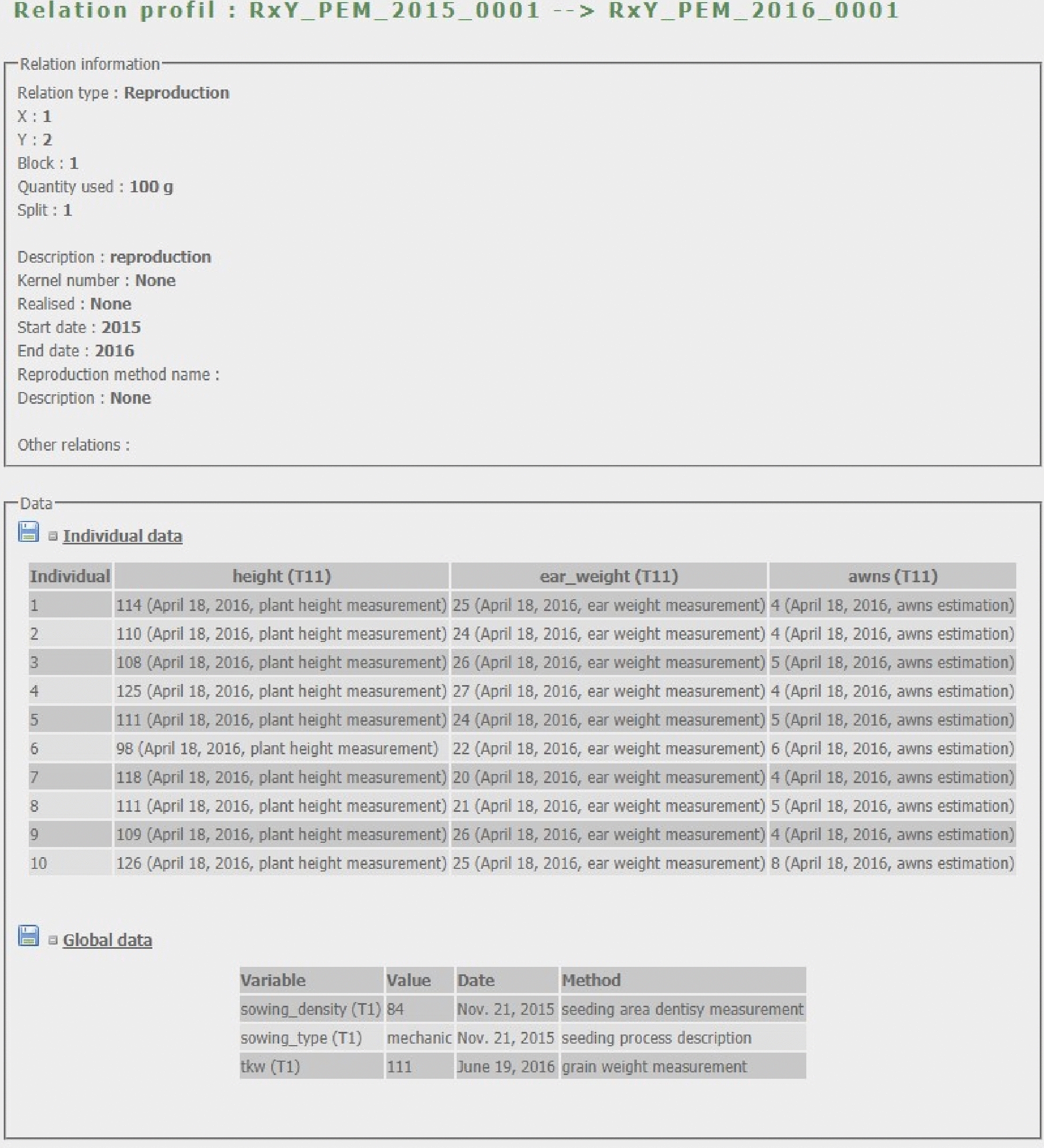
Relation card. The relation card is divided in two frames: (1) The information about this relation. (2) The data related to this relation at global and individual level

From any query result, the user can access the seed lot card (Fig. 4). It displays information about the seed lot (its name, stock location, creation year, germplasm etc.), how it was created and used and its stock evolution. The stock is computed using initial quantity of the lot, all quantities used in relations as a parent and the inventories of the stock. The user can also access to the relationship card (Fig. 5) between two seed lots. It displays all the data that has been measured for this event and the associated seed lots. From any card the data tables can be downloaded in tabulated text file.

Lastly, SHiNeMaS provides a global search bar enabling a quick access to the seed lots, relations or germplasms cards. This search bar is implemented with an auto-complete feature.

### Support and community

SHiNeMaS source code is hosted on the French national forge for research and higher education, SourceSup^3^. Through this forge, our aim is to provide tools to the community such as an issue tracker, forums or wiki site. This forge provides anyone who wants to contribute to the development of our project the usual tools of popular forges.

Currently, the web interfaces of SHiNeMaS are available in English or French but, with the help of the community, it would be easy to translate the application in any other language. A virtual machine is also available to run SHiNeMaS’ tutorial^4^.

### Future developments

Major future developments of SHiNeMaS will focus on the management of new data types (climatic data and plant pictures). In addition to storing data on seed lots and relations between seed lots, it will be possible to store data on germplasm. A web service will be developed to provide features ensuring interoperability with external tools such as R packages [7]. Efforts will also be made in terms of controlled vocabulary by complying with the Plant Science community of Elixir^5^ and we aim to implement methods of the Breeding API^6^. The version 2.0 of this API defines new methods related to seed lot and seed lot transactions which would be relevant for our tool. Moreover, we will ensure an audit of submitted files to keep track of the submissions. In a future version of SHiNeMaS, we will focus on the use of controlled vocabulary and ensure the interoperability with standard ontologies. Using the same user role for database administration and data management can be a risk if the data manager is not aware of all these privileges. To fill this gap we will improve the data access control and define more user roles in a further SHiNeMaS release.

## Conclusion

We developed SHiNeMaS to ensure seed lots traceability and to manage phenotypes, environments and cultural practices data for various genetic resources, varieties of many crop species. This web application is a key feature as it enables data administration at different scales (files, or single forms). SHiNeMaS is especially adapted for field experimentation in a network of farms or experimental stations and that requires tracking seed lots history. It is available under AGPL licence and uses free technologies. SHiNeMaS has been used in production in our laboratory for 5 years and some farmers’ organizations facilitators manage their own information in the system.

## Supplementary information

**Additional file 1.** Database schema. Relational schema of SHiNeMaS’ database.

## Data Availability

Project name: SHiNeMaS. Project home page: https://sourcesup.renater.fr/projects/shinemas. Operating system(s): Platform independent. Programming language: Python. Other requirements: Python3, Django 2.0. License: Affero GPL. Any restrictions to use by non-academics: None.

## Footnotes

1 https://www.djangoproject.com/.

2 https://www.iso.org/iso-8601-date-and-time-format.html.

3 https://sourcesup.renater.fr/.

4 https://sourcesup.renater.fr/wiki/shinemas/en:vmtuto.

5 https://elixir-europe.org/communities/plant-sciences.

6 https://www.brapi.org/.
