# Supplementary figures and images for: SHiNeMaS: a web tool dedicated to seed lots history, phenotyping and cultural practices
Source: Plant Methods. 2020 Jul 23;16:98. doi: 10.1186/s13007-020-00640-2 (PMC7376679; doi:10.1186/s13007-020-00640-2)

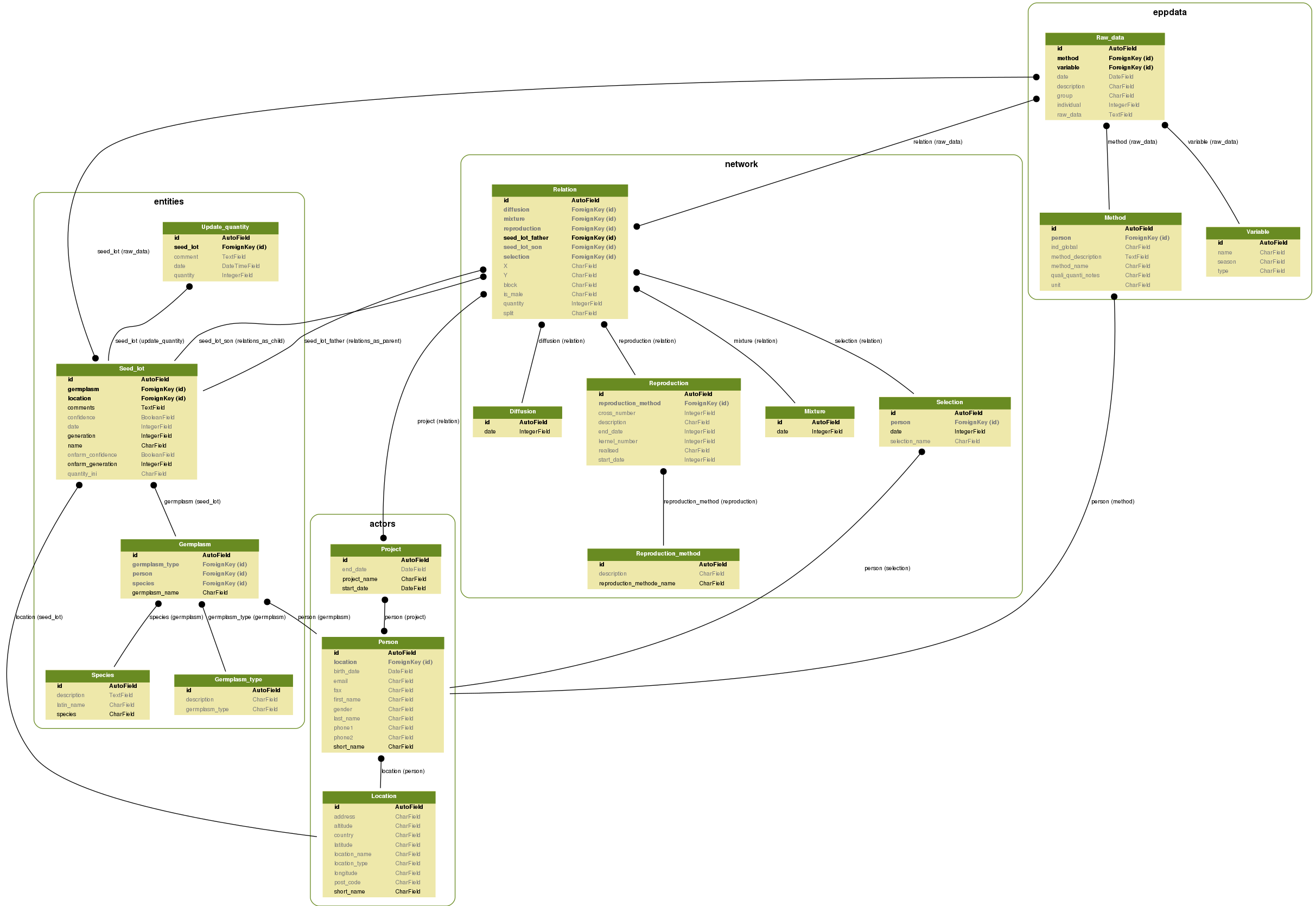

Supplement: Supplementary file 1 — Additional file 1. Database schema. Relational schema of SHiNeMaS’ database. [file 13007_2020_640_MOESM1_ESM.png]
